# A pathogen-specific approach towards udder health management in dairy herds: Using culture and somatic cell counts from routine herd investigations

**DOI:** 10.4102/ojvr.v83i1.1146

**Published:** 2016-08-30

**Authors:** Inge-Marié Petzer, Joanne Karzis, Edward F. Donkin, Edward C. Webb

**Affiliations:** 1 Department of Production Animal Studies, University of Pretoria, South Africa; 2 Department of Animal and Wildlife Sciences, University of Pretoria, South Africa

## Abstract

A dedicated udder health diagnostic programme was developed and used over a 15-year period in South Africa to analyse milk samples based on microbiological and cytological patterns within various groups and for individual cows and udder quarters in dairy herds. These pathogen-specific analyses are utilised for pro-active improvement and management of udder health in South African commercial dairy herds. The programme acts as a monitoring tool and identifies management areas at risk and individual cows with udder disease and uses both quarter and composite milk samples. Intra-mammary infection (IMI) is a dynamic situation and depending on the time a milk sample is taken, false-negative results may be obtained. A new IMI and an infection that is curing may both have low somatic cell counts (SCCs), masking the true bacterial status. SCC in individual infected udder quarters may differ greatly depending on the causative bacterial species, its pathogenicity, the host immune status and the environmental factors involved. A pathogen-specific udder health approach was followed with repeated herd tests to take account of these udder health dynamics. The results of the herd IMI investigation are applied in practice to assist veterinarians, udder health consultants and managers to make informed and specific detailed decisions at both a herd and on an individual cow basis regarding udder health.

## Introduction

Mastitis is an endemic disease and is considered the most frequent and costly disease in the dairy industry responsible for the highest financial losses, which affects both the animal and the quality of the product (Halasa *et al*. 2007; Hogeveen *et al*. 2010).

Mastitis is complex and multifactorial in nature and generally results from an interaction between a variety of microbial infections, host factors and environmental and management factors and with generally poor treatment success. It is defined by the National Mastitis Council (NMC) as an inflammation of the mammary gland mainly caused by bacterial infection (NMC Guidelines 2001). Dealing with clinical mastitis cases remains important but damage to the udder parenchyma may lower a cow’s lifetime production potential and increases the risk of infecting fellow cows by the shedding of pathogens (DeGraves & Fetrow 1993; White 2010). Optimal management practices are considered to be the most effective way to control the disease. Work done during the 1960s set the stage for our current understanding of mastitis and established standards for contagious mastitis control (Davidson 1961; Dodd *et al*. 1969; Neave, Dodd & Kingwill 1966; Neave *et al*. 1969). From this work, a five-point mastitis control programme was developed and was later upgraded to the NMC 10-point mastitis control plan (Smith & Hogan 2001). Utilisation of this programme has led to a reduction in the prevalence and elimination of contagious mastitis from many South African farms. However, *Staphylococcus aureus* and *Streptococcus agalactiae* remain a challenge in individual farms in South Africa and abroad (Petzer *et al*. 2009).

In a paper published by Middleton (2013), he questioned what we have learned regarding *S. aureus* mastitis in the last 50 years. His answer summarised the global udder health dilemma. We have gained much knowledge but the basic fact that milking time hygiene is the main critical control point has not changed. Decisions regarding the control of mastitis in a given herd will depend on the contagiousness, persistence and inflammatory nature of the main infecting species and strains. The use of historical data to evaluate true new intra-mammary infection (IMI) rates, the extent of chronic carriers within the herd and the bacterial cure rates in combination with somatic cell count (SCC) levels are all valuable measures to identify mastitis and stress-related causes of high SCC.

Knowledge of the bacterial species present in the udders of cows in a herd and the ability to identify cows with and without IMI can be used as a tool for in-depth management decisions. In order to control the transmission rate of contagious pathogens by limiting new IMI, cows infected with contagious udder pathogens can be milked last, thereby limiting the risk of spreading this infection to the healthy cows. Parlour supervision of the cows infected with contagious pathogens should then be intensified. It has been well established that the probability of IMI cure depends on the cow, pathogen and treatment factors (Barkema, Schukken & Zadoks 2006). Although treatment success is influenced by choice and use of an appropriate product and also the lactation stage of the cow, the main factor influencing treatment success has been found to be treatment duration (Barkema *et al*. 2006). In addition to the bacterial species or strains involved, their pathogenicity and possible antimicrobial resistance need to be considered, as well as the immune response of the cow. Cure rates for *S. aureus* mastitis have been found to range from 3 to 74%. Cure is lower in older cows, and in those with high SCC, it increased chronic IMI and increased the numbers of mammary quarters infected (Barkema *et al*. 2006). Spontaneous cure of new *S. aureus* IMI may be as high as 21%, while in chronic cases, this figure can be as low as 3% (Swinkels, Schukken & Cox 2012). Only a few udder pathogens such as *S. agalactiae* are known for a high treatment success during lactation (Keefe 1997). *Staphylococcus aureus* and *S. agalactiae* IMI are still present in dairy herds in South Africa (Petzer *et al*. 2013) and warrant a more efficient testing system to enable producers to eliminate both these pathogens from their herds.

The SCC measure has been used worldwide for several decades as a primary indicator of udder health in dairy herds (Heeschen 2010; Hillerton 1999; Reneau 2001). More than 95% of the cells in milk are leucocytes consisting of variable proportions of macrophages that recruit the neutrophils and lymphocytes in the event of invading pathogens by releasing chemo-attractants (Burvenich *et al*. 2000; Sharma, Singhand & Bhadwa 2011; Zeconni & Smith 2000). The lymphocytes act mostly as memory cells for the immune system while neutrophils phagocytise and destroy the pathogens. Less than 5% of SCC consists of epithelial cells originating from the mammary gland (Harmon 2001; Lee, Wooding & Kemp 1980). The level of SCC in milk depends on various factors of which IMI is the most important. Factors include parity, stage of lactation, milking frequency, stress (environmental, nutritional, systemic disease and day-to-day changes in management) and non-specific disturbance (NSD) where an increase in SCC is not caused by pathogens (Laevens *et al*. 1997; Sandrucci *et al*. 2014; Wegner *et al*. 1976; Yagi *et al*. 2004).

Traditionally, bulk milk tank somatic cell count (BMSCC) is used as a primary index when analysing herd udder health and is used as one of the quality criteria for payment by the secondary milk industry (Ruegg & Pantoja 2013). High BMSCC could indicate udder health problems, whereas a too low level (below 100 000 cells/mL) should act as a warning that mastitis caused by coliform bacteria may increase (Shuster, Lee & Kehrli 1996; Suriyasathaporn *et al*. 2000). However, the BMSCC provides only an estimation of the prevalence of infection and irritation of the udders of the cows. This is not a true reflection of the herd udder health status as milk from problem cows should have been excluded (cows with clinical mastitis, fresh in milk cows, cows under treatment and sometimes those with high SCC). The BMSCC can also be influenced by herd dynamics such as the average parity of cows, and the distribution of lactation stage seen in seasonality calving herds compared to herd with an all-year-round calving pattern (Laevens *et al*. 1997). The impact of any individual cow on BMSCC will further depend on its SCC and the milk yield of the cow and the dilution factor within the tank. It has been estimated that there is likely to be a 10% increase in IMI prevalence in a herd for every 100 000 cells/mL increase in BMSCC (Bradley 2007). In the year 2014, an estimated 145 million dairy farms operated globally and the largest average dairy herds were found in Saudi Arabia (8125 cows) followed by New Zealand (393 cows) and South Africa (238 cows) (Lactodata 2014). The larger the herd, the more critical the interpretation of the BMSCC needs to be and the less valuable it becomes as a pro-active udder health monitoring tool because of the dilution effect of the milk masking high SCC of an individual cow.

To evaluate changes in udder health status, the SCCs of individual cows are often analysed together with herd clinical mastitis statistics (Bradley & Green 2001). An SCC threshold is used as a guideline to estimate new IMI, chronic IMI and cure rates and those cows or udder quarters that remain without IMI. There has never been absolute consensus regarding the correct threshold level to indicate IMI. Some consensus was reached regarding an SCC standard of ≥ 200 000 cells/mL quarters milk as the indicator of IMI at two World Dairy Summits (Athens 1999; New Zealand 2001). However, it was agreed at these summits that a tolerance range of up to 400 000 cells/mL was necessary for practical reasons (Heeschen 2010). Regardless of the international guidelines, countries still set their own SCC thresholds, indicating that there is still no consensus at the operational level. The reliability of records of clinical mastitis cases depends on accurate detection of mastitis on a day-to-day basis on farms, and these records are often questionable.

The aim of this article is to outline a fresh approach to herd udder health monitoring that facilitates an in-depth analysis of clinical and subclinical mastitis using software that has been developed and tested over the past 15 years at the Faculty of Veterinary Science, University of Pretoria. Currently, more than 930 commercial dairy herds from South Africa and neighbouring countries are benefiting from its application. Sample analysis is paid for by the dairy producers, but it is partially subsidised by the University of Pretoria to keep costs for whole herd analysis reasonable. In return, the University gains current udder health data that can be utilised for pre- and postgraduate student training and research. This data set provides current and historical data for research and contains at present results of over 1.6 million milk samples. The Milk Sample Diagnostic (MSD) programme that was developed provides an overview of the current and historical herd udder health situation and allows for analysis related to parity, days in milk and other herd groupings and facilitates decision making on an individual cow level based on current and historic species-specific and udder pathology information. It is used as a practical tool to identify challenges at the herd, cow and udder quarter level and guides decision making at an operational level. Only by addressing the particular cause can the problem be eliminated. This udder health approach differs from the conventional approaches that use primarily SCC and data of clinical mastitis cases as an aid to decision making.

## Materials and methods

### Milk samples and data

Udder quarter and composite milk samples (one milk sample taken from all four quarters) are taken in most cases from all lactating cows in a herd to establish the presence and prevalence of udder pathogens. Professional milk samplers assist milk producers in South Africa to take samples in an aseptic manner, ensuring that the sample quality is good and that the cold chain is maintained until the samples reach the Milk Laboratory. Most milk samples reach the laboratory within 24 hours. On arrival at the Milk Laboratory (Department of Production Animal Studies, Faculty of Veterinary Science, University of Pretoria, South Africa), the batch temperature of the milk samples is recorded. Milk is then plated out on Bovine Tryptose agar, and cultures are read after 18–24 and 48 h (Petzer *et al*. 2012). SCCs were performed using a Fossomatic 5000 (Petzer *et al*. 2012). Identification of mastitis pathogens is generally completed within 48–72 h after the samples have been received at the laboratory.

In addition to SCC, culture results, clinical appearance of milk and the general information about the cow are added to the MSD programme. This includes information regarding calving dates, parity, milk yield, stage of pregnancy, status of the udder parenchyma (assessed by palpation) and teat canal scores.

Results of quarter milk and composite milk samples are analysed separately and are summarised in individual cow reports and various group reports.

Some of the various reports that will be discussed:

- The Serial Herd Udder Health Report used for evaluation of composite cow milk samples provides an overview of the current and historical udder health status of the herd based on microbiological and SCC results. The report also provides a perspective on the level of new, persistent and cured cases for each bacterial species isolated from the herds and indicates the SCC distribution within the herd.
- A Current Herd Udder Health Report for analysis of quarter milk samples summarises the SCC and culture results of the current test of the herd as percentage of quarters with mastitis, or with NSD, IMI with low SCC, and the percentage of normal quarters.
- Group Reports focus on species identification, early post-partum reports (for the first 30 days in lactation) and a lactation stage report (5–90, 91–180 and ≥ 180-day groups) also differentiating between parities.
- The Economic Report provides financial information on probable loss in revenue as a result of milk not being produced because of an udder that is not completely healthy, indicated by the SCC level.

### Criteria used for diagnosis

Microbiology (culture) results and SCCs are available for each quarter milk sample. Many countries set their own operational SCC threshold that is in accordance with recommendations of the World Dairy Summits of less than 400 000 cells/mL. An SCC threshold of 300 000 was there for chosen for this programme, being midway between the 200 000 cells/mL recommended by the International Dairy Federation (IDF) and the practical threshold of 400 000 cells/mL recommended at the World Dairy Summit. In this programme, quarters that tested bacteria negative with an SCC of below 300 000 cells/mL milk were regarded as being normal (N); quarters that tested bacteria positive with an SCC of equal or above 300 000 cells/mL milk were regarded as having mastitis (M); quarters that tested bacteria positive with an SCC of below 300 000 cells/mL were identified as having a ‘teat canal infection’ (TCI); and quarters that tested bacteria negative with an SCC of equal or above 300 000 cells/mL were identified as having an NSD. The desired herd values aimed for are less than 5% of quarters with mastitis, less than 5% quarters with TCI, less than 3% quarters with NSD and more than 50% normal quarters (Giesecke, Du Preez & Petzer 1994).

## Results and discussions

### Serial herd health report (composite milk samples)

The Serial Herd Udder Health Report is presented in two parts. The first part provides a summary overview of one to four consecutive herd examinations based on SCC and culture results of composite milk samples, while the second part deals with the SCC. The current herd udder health status is analysed and compared to results of previous examinations. Positive progress or negative developments in the herd in terms of IMI and SCC trends is measured and evaluated. The various bacterial species isolated from individual cows are indicated as numbers and percentages of cows sampled (see Table 1) while the SCC are summarised according to six threshold levels (Table 2).

**TABLE 1:** Serial Herd Udder Health Report. Case study Part 1: Bacteriology history report for four consecutive herd examinations of the same herd using composite cow milk samples.

Bacteria isolated	Diagnosis	Numbers examined							

		28 October 2014		17 November 2014		04 June 2015		02 July 2015	
			
		1290	%	1304	%	1366	%	1297	%
*Staphylococcus aureus*	Total	78	6.05	214	16.41	72	5.27	58	4.47
	New	78	6.05	159	12.19	22	1.61	12	0.93
	Repeat	0	0.00	44	3.37	32	2.34	27	2.08
	Cured	0	0.00	11	0.84	18	1.32	19	1.47
*Streptococcus agalactiae*	Total	24	1.86	0	0.00	73	5.34	45	3.47
	New	24	1.86	0	0.00	72	5.27	40	3.08
	Repeat	0	0.00	0	0.00	1	0.07	5	0.39
	Cured	0	0.00	1	0.80	8	0.59	33	2.54
*Streptococcus dysgalactiae*	Total	4	0.31	0	0.00	2	0.15	2	0.15
	New	4	0.31	0	0.00	2	0.15	1	0.80
	Repeat	0	0.00	0	0.00	0	0.00	1	0.80
	Cured	0	0.00	3	0.23	1	0.70	1	0.80

**TABLE 2:** Serial Herd Udder Health Report. Part 2: Somatic cell count history report for four consecutive herd examinations of the same herd based on the results of composite cow milk samples.

SCC × 1000 cells/mL	Dates × Values (% of lactating cows)							

	28 October 2014: 1290 cows		17 November 2014: 1304 cows		04 June 2015: 1366 cows		02 July 2015: 1297 cows	
			
	%	Cum %	%	Cum %	%	Cum %	%	Cum %
1–125	35.71	35.71	25.14	25.14	36.85	36.85	59.46	59.46
126–250	13.75	49.46	15.45	40.62	17.61	54.46	9.27	68.73
251–375	9.53	58.99	11.48	52.09	10.56	65.02	5.66	74.39
376–500	6.39	65.38	7.29	59.38	5.87	70.89	3.99	78.38
501–750	8.32	73.70	9.49	68.87	7.51	78.40	4.89	83.27
≥ 750	26.30	100.00	31.15	100.00	21.60	100.00	16.73	100.00

Cum, Cumulative.

### Part 1: Serial Herd Microbiological report

Each bacterial species isolated from individual composite milk samples in the herd is indicated as a number and as a percentage of the total samples examined. Of each bacterial species isolated, the number of new, repeat or persistent cases and cases cured are indicated. In the first examination of a herd, all cases are indicated as new infections. In consecutive herd examinations, new infections are indicated when a specific bacterial species has not been isolated from the same cow at the previous examination; persistent cases are those with identical bacterial isolations as shown previously; and ‘cases cured’ (those that are culture negative) in the current examination for the specific bacterial species were those that were isolated during the previous examination (Table 1).

#### Prevalence of species-specific herd intra-mammary infections

Depending on the principal bacteria present and its prevalence in the herd, management strategies should be planned in collaboration with the herd manager. A policy of zero tolerance is followed in most cases where *S. agalactiae* and *S. aureus* are isolated, aiming at the eradication of these bacteria from all udders in these herds. This approach has proved to be practical and successful in South African herds over the past 15 years. The prevalence of *S. aureus* IMI in more than 930 South African commercial dairy herds decreased from 14.08% in 2008 to 7.77% in April 2012 (Petzer & Karzis 2012) and to 5.14% in December 2014 (Petzer, unpublished data). Bacteriological results as shown in Table 1 provide the veterinarian, udder health consultant and dairy manager and owner with detailed results to assist informed decision making regarding udder health management at the herd level.

#### New intra-mammary infections

Depending on the species of bacteria and the level of new IMIs in the herd, deductions can be made regarding parlour hygiene and bedding management (Table 1). Management may prove to be inadequate and protocols may need to be revised and upgraded.

The level of new IMI can be a valuable measurement of effective parlour hygiene and of milker education, dedication and health, especially in the case of contagious mastitis bacteria such as *S. aureus* and *S. agalactiae*. It can also indicate ineffective separation of *S. aureus*– and *S. agalactiae*–positive cows or an incorrect milking order. The level of new infections may increase if there is a lack of biosecurity when new cows or heifers are introduced into a dairy herd without determining the status of their IMI prior to allowing them on the farm, in the parlour and mixing them with the local herd. Bacteria such as *S. aureus* and *Streptococcus pyogenes* are known to be able to cause reverse zoonosis (Messenger, Barnes & Gray 2014). When reverse zoonosis is suspected in a herd, milkers and people in close contact with the cows should be tested for the presence of these bacteria by requesting throat swabs.

When most new IMI are predominantly environmental bacteria such as the coliforms (*Escherichia coli, Klebsiella* spp. and *Serratia* spp.) or streptococci other than *S. agalactiae,* then udder, feet and flank hygiene scores (Cook & Reinemann 2007) can be performed to quantify the challenge and identify areas of risk. Sources may include inadequate management of bedding, which can cause increased levels of loose faeces on bedding surfaces (Reneau *et al*. 2003), water pollution (mineral or microbial) or overcrowding.

#### Repeat (persistent) cases and cases cured

When the same bacterial species is isolated from the same udder (composite milk samples) or the same quarter (quarter milk samples) on two consecutive examinations within a reasonable time period, it is regarded as a repeat or persistent IMI. The percentage of cases that repeat and those cured (bacterial cure) indicate the level of chronicity and the problematic bacterial species or strains present in the herd. It may be an indication of ineffective mastitis treatment. Udders of cows that repeat are palpated to identify possible parenchyma pathology because unsuccessful treatment may be as a result of fibrosis, nodules or atrophy of the udder parenchyma and not necessarily because of bacteria that are resistant to antimicrobials. ‘Fibrosis’ (hardening udder quarter) is used as an indication of a more recent chronic case compared to ‘atrophy’ (shrinking udder quarter).

#### Application of results in a *Streptococcus agalactiae*–positive herd

The herd indicated in Table 1 had 3.47% of cows infected with *S. agalactiae* at the examination dated 02 July 2015. Although this percentage decreased from the previous examination on 04 June 2015 (from 5.34% to 3.47%), too many (40 of 45) of these infections were new *S. agalactiae* IMI possibly indicating a relaxed parlour hygiene. Five cases of *S. agalactiae* IMI persisted and 33 had been cured since the June examination. The producer was asked to provide information on the whereabouts of 35 cows that were infected with *S. agalactiae* in June 2015 and which were not tested in July. These cows might have been dried off, removed from the herd or were merely not sampled. The target for new IMI and persistent cases caused by *S. agalactiae* should both be < 5%. A revised management plan should include better prevention and follow-up of cases that did not cure.

In this case where *S. agalactiae* IMI has been isolated from a herd a partial ‘blitz therapy’ can be implemented because in this case positive cows have been identified. They should immediately, after conformation of their infections status, be separated from the rest of the cows for the treatment period and until they have been resampled and found to be cured (bacteria free). Retesting of both the lactating herd and treated cows is essential for the successful elimination of *S. agalactiae* from the herd in a relative short period of time. A percentage of *S. agalactiae*–infected cows may have been missed during the laboratory examination because of the small volume of milk plated out; or because of sub-minimal concentrations of bacteria present in milk samples; or because of the presence of coagulase-negative staphylococci (CNS) in udders with high SCC initially masking the presence *S. agalactiae* (personal experience). A short laboratory turnover time in the case of *S. agalactiae* IMI is crucial to the success of eliminating these bacteria from the herd (Keefe 1997).

#### Case study: Application of results in a *Staphylococcus aureus*–positive herd

In herds where a low prevalence of *S. aureus* is identified, the few positive animals should be culled as soon as possible, and management should focus on sound parlour and milking procedure hygiene. The lactating herd should be retested and quarter udder secretion samples of dry cows in late gestation should be included. When a herd is identified with a medium to high prevalence of *S. aureus*, a longer term strategy is adopted rather than that of culling all positive animals, although culling will form part of the action taken. An important action will be to upgrade the protocol, application of parlour management, the milking routine and the hygiene and monitoring strategies. Other factors such as the within-herd prevalence, the contagiousness of the bacteria, the milk price, the current percentage of cows culled because of mastitis and the number of replacement heifers available must also be considered (Bradley 2007).

The herd indicated in Table 1 was diagnosed with 78 (6.05%) *S. aureus* cows in October 2014 and was regarded as herd with a moderate level of infection prevalence. The producer would be advised to separate the *S. aureus*–positive cows, if possible for life, and to keep them in a *S. aureus* group. The results from November 2014 (Table 1) showed an increased in the number of *S. aureus* cases to 214 (16.41%). Of these, 159 cows (12.19%) had new *S. aureus* infections indicating that the preventative measures were inadequate, 44 cows (3.37%) showed persistent infection from the previous test, indicating in these cases a high possibility of chronic cases and only 11 cows (0.84%) were apparently ‘cured’.

**FIGURE 1:**
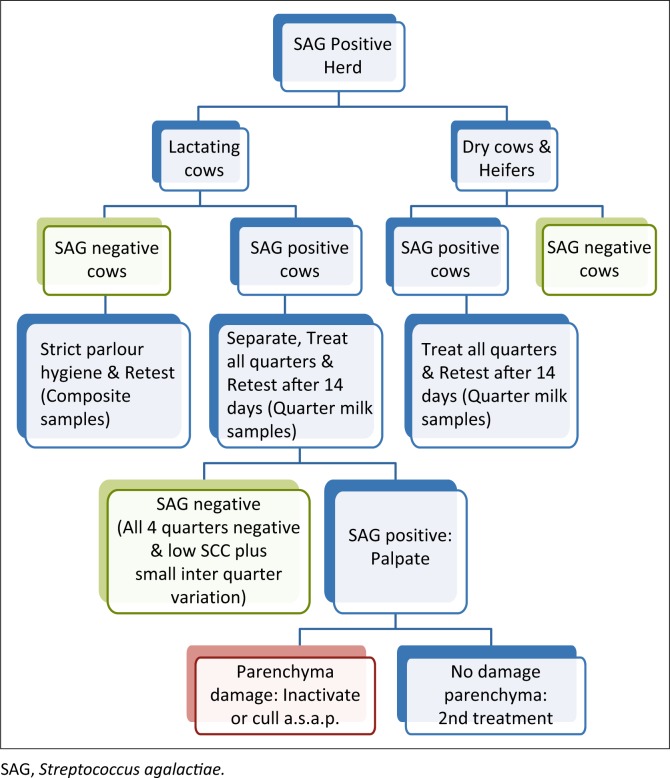
A flow chart indicating events during the management of a Streptococcus agalactiae intra-mammary infection outbreak in a dairy herd.

Though the choice and duration of treatment should be discussed with the farmer, the probability of cure could be calculated for each *S. aureus–*positive cow using the system developed by Sol *et al*. (1997). This formula incorporates parity (first lactation and higher), stage of lactation (early, mid or late), level of SCC (above or below a linear score of 6.9 or approximately 800 000 cells/mL), the number of quarters per udder positive for *S. aureus* (less or more than 3) and quarter position (front or hind) of individual cows, as well as the treatment duration. It can be used to calculate the probability of *S. aureus* cure (Swinkels *et al*. 2012) (Figure 1). Information for individual cows on parity, lactation stage, pregnancy status, milk yield, mastitis and SCC history is available in the MSD programme to aid in the decision making. Udders of *S. aureus* cows should be palpated to identify gross parenchyma damage such as fibrosis, nodules and atrophy, which usually would make treatment ineffective. An informed decision could then be made regarding actions to be taken in case of each individual *S. aureus* cow, and a detailed action list for individual cows can be formulated for the manager. This might include intra-mammary therapy with or without an extended duration, early drying-off with therapy, inactivation of a quarter or culling of the cow.

In this particular herd (Table 1) at the next examination date on 04 June 2015, the number of new cases had decreased to 22 cows (1.61%) of samples, while persistent infections remained relatively low at 32 (2.34%), as does the numbers with bacterial cure (1.32%). However, Staphylococcus ‘cure cases’ need to be retested because of the nature of *S. aureus* to shed intermittently. This is the reason why these cows should remain separate from the rest of the herd for life.

South African dairy managers and veterinarians are aware of the existence of reverse zoonosis where infections carried by people may pose a threat to udder health of cows. A noteworthy number of the work force (Statistics South Africa 2013) has immune systems compromised because of HIV with a consequence of an increased risk for disease. Milkers with upper respiratory tract infections caused by *S. aureus* may pose a risk to the udder health of dairy cows if hygienic principles are not adhered to in the parlour (Petzer *et al*. 2009). Precautions such as the wearing of facial masks and placing milkers that have tested positive for *S. aureus* infections into strategic milking positions where they do not need to touch udders (like teat dipping) have been helpful (personal experience).

#### Application of results in herds with mainly gram-negative Intra-mammary infection

When IMI with bacteria of environmental origin is predominant in a herd, the management focus should shift to camps, pasture, bedding and the parlour as sources and areas of risk. The specific bacterial species may provide information on the probable source. *Pseudomonas* spp. for instance is often found in a water source, and *Listeria* spp. is mostly present in silage (Hogan & Smith 2003). In South African pasture–based dairy herds, the prevalence of *Streptococcus uberis* is increasing (Petzer *et al*. 2009).

### Part 2: Serial herd somatic cell count report

In the second part of the Serial Herd Udder Health Report, six SCC thresholds are calculated from the herd test (percentages and cumulative percentages). The SCC increments are 125 000 cells/mL up to 500 000 cells/mL and become larger for higher SCC levels. The herd target is to have more than 80% of lactating cows with SCC of less than 250 000 cells/mL, while less than 5% should have an SCC in excess of 750 000 cells/mL. Herds with a high percentage (> 90%) of cows with low SCC may be at a higher risk of contracting *E. coli* mastitis. In such a situation, management practices such as teatdip prior to milking and feeding immediately after milking to prevent cows from lying down would be advised to allow enough time for the teat canal to close. This is especially true of high-yielding herds where the immune system of cows is more likely to be weakened during the first trimester of their lactation (Hogan & Smith 2003).

SCC dynamics of four consecutive herd investigations from 26 October 2014 to 02 July 2015 are compared in Table 2. The percentage of cows with SCC below 250 000 cells/mL is still low in July 2015 (68.13%), although it has improved from 40.62% in November 2014. Similarly, the percentage of cows with SCC above 750 000 cells/mL decreased from 31.15% to 16.73% for the same period but is not yet on target.

In herds with a high proportion of cows with high SCC, a distinction is made between those with and without IMI. In cases where most samples that showed high SCC also had IMI, the sources of these infections should be identified and eliminated or managed. When samples from which no bacteria were isolated form a significant portion of samples with high SCC, possible stressors and causes of udder irritation need to be investigated. Composite milk samples from cows identified with high SCC can be tested on farm with the California Milk Cell test to gain insight into the inter-quarter SCC relation. Heat stress, mud stress, nutritional stress and social or handling stress are examples of stressors that can be responsible for increased SCC (Du Preez 2000). Differences in SCC between quarters of the same udder are used. Physiological changes and stressors to the cow will be more prone to cause an elevated SCC in three or four quarters, while udder irritation is more often seen in only one or two quarters of an udder. The latter can be caused by incorrect milking techniques, incorrect milking machine settings or lack of adequate machine maintenance. Cows that have recently been treated with intra-mammary antimicrobials could also test culture negative with a high SCC.

The probability of incorrect milking machine settings and incorrect use causing irritation can be investigated by using teat canal scoring (Neijenhuis *et al*. 2001) on first lactating cows, 1–3 months into lactation. The teat canal is the first line of udder defence and a very important barrier preventing IMI when damaged. Pulsator function should be tested by performing the static test on all milking units followed by dynamic testing to check vacuum stability and level at the teat end during milking. Milking machines with high milk lines as well as swing-over parlour systems are still used in South African dairies. Teat canal damage is more likely occur in high–milk lines systems than in low milk lines because when over-milking occurs, the risk of a high–teat-end vacuum is greater in high-line systems that function at a higher system vacuum. Too high–teat-end vacuum is known to be responsible for teat-end damage (Reinemann *et al*. 2008). Swing-over systems with automatic cluster removers are now installed in South African dairies. Flow metres that measure the milk flow per unit and initiate cluster take-off are installed in these systems far above the level of the udder, and the milk lines transporting milk from the cluster to the flow metres are often more than 1.5 m – 2.0 m long. Therefore, it is too long a delay in the time from when the take-off flow rate is reached until the cluster is actually removed, increasing the risk of over-milking.

Milking routine should be monitored on site, and a System Lactocorder (WMB AG, Balgach) can provide measurements of the milk let down time, rate of milk flow and the timing of cluster removal. Automated inline monitoring systems such as Afimilk programme (Afimilk Ltd, Kibbutz Afikim, Israel) are available to identify cows that are in incorrect groups and to identify current and past trends in the milking routine. These can include the time from touch to attachment of clusters, milking speed, cluster fall-off, re-attachment and early detachments. Reasons for insufficient stimulation or delay in take-off can be identified and rectified, whether by training or notifying the milkers or by correcting a milking machine fault.

Nutritional stress can contribute to high SCC and practical methods such as bunker score and space (Bolsen & Pollard 2004), rumen filling (Burfeind *et al*. 2010), faecal score and percentage of cows ruminating may be used as a starting point for the investigation in total mixed-ration herds, followed by an in-depth analysis of the feed when indicated. Cows on pastures and in paddocks may suffer from stress because of mud during the rainy season. In South Africa, the high Temperature Humidity Index (THI) may often have a negative effect on the SCC levels, milk yield and reproduction efficiency during the hot summer months (Du Preez, Giesecke & Hattingh 1990; Giesecke *et al*. 1988).

### Current herd udder health report (Quarter milk samples)

A summary of species-specific IMI and SCCs for herds is shown in Table 3. The report is divided into two sections with the first part indicating results from lactating cows and the second part from non-lactating cows. The MSD programme currently uses an SCC level of 300 000 cells/mL in quarter milk samples as threshold to diagnose mastitis when IMI is present and NSD in the absence of IMI. Quarters with an SCC level below 300 000 cells/mL without IMI are diagnosed as ‘normal’ and those with IMI as ‘TCIs’. Dry cow secretions are only examined for the presence of bacteria and no SCC is done.

**TABLE 3:** Current Herd Udder Health Report based on somatic cell count and culture results of quarter milk samples from lactating and dry cows.

Cows	Diagnosis	Micro-organisms	Right front quarters (%)	Right hind quarters (%)	Left front quarters (%)	Left hind quarters (%)	Total (%)
Lactating	Mastitis	*Staphylococcus aureus*	0.0	1.1	1.1	1.1	3.3
	Mastitis	CNS	0.0	2.1	0.0	0.0	2.1
	Mastitis	*Streptococcus uberis*	0.0	0.0	0.0	1.1	1.1
	Normal	None	14.7	10.1	9.8	10.9	45.5
	NSD*	None	5.9	8.4	6.4	8.6	29.3
	TCI**	*Staphylococcus aureus*	3.3	1.8	2.2	0.0	7.3
	TCI	CNS	1.1	1.5	4.4	3.3	10.3
	TCI	*Streptococcus agalactiae*	0.0	0.0	1.1	0.0	1.1
Dry	Dry Normal	None	8.0	12.0	8.0	17.0	45.0
	Dry IMI	*Staphylococcus aureus*	5.0	3.0	3.0	1.0	12.0
	Dry IMI	CNS	10.0	8.0	11.0	5.0	34.0
	Dry IMI	*Streptococcus uberis*	2.0	2.0	3.0	2.0	9.0

Mastitis criteria: SCC ≥ 300 000 cells/mL and culture positive; normal criteria: SCC < 300 000 cells/mL and culture negative.

* NSD: SCC ≥ 300 000 cells/mL and culture negative;

** TCI: SCC < 300 000 cells/mL and culture positive.

CNS, coagulase-negative staphylococci; NSD, non-specific disturbance or udder irritation; TCI, teat canal infection; IMI, intra-mammary infection; SCC, somatic cell count.

Two major concerns can be identified regarding the udder health status in the lactating cows in the herd indicated in Table 3, namely the large percentage of quarters with high SCC and the presence of *S. aureus* IMI in the herd. *Staphylococcus aureus* was isolated from 10.6% of quarters, CNS from 12.4% and *S. uberis* and *S. agalactiae* each from 1.1%. Of the 35.9% quarters with SCC of 300 000 cells/mL and above (mastitis and NSD cases), only 6.6% had IMI, indicating that something besides IMI was prime reason causing high SCC in the herd, even though 25.3% of all quarters had IMI. More hind than front quarters were diagnosed with NSD and might indicate incorrect removal of clusters.

Of the dry cows sampled 4 weeks after intra-mammary treatment, 12% were infected with *S. aureus*, 34% with CNS and 9% with *S. uberis*. This could indicate a poor cure rate for *S. aureus* and a possible high new IMI occurring during the dry period for both CNS and *S. uberis* when compared to the results of the lactating cows (Table 3).

An action list could be compiled for the producer which may include: *S. aureus* cows to be followed up by evaluating their mastitis history, performing udder palpation and evaluating criteria of each cow for her probability of cure. The milking machine should be checked, milk routine evaluated and other possible stressors investigated.

### Group reports

Calving dates, pregnancy status, level of milk yield and status of the udder parenchyma can be entered into the MSD programme as information additional to the laboratory results for individual cows.

#### Pathogen-specific group report

Reports of cows currently infected with specific udder pathogens are generated to use as on-farm action lists. As explained above, cows should be selected and separated, to deal with contagious IMI such as *S. aureus* or *S. agalactiae* IMI in a herd. Cows that have had *S. agalactiae* IMI and were cured (bacterial cure) may be returned to their previous groups, but *S. aureus* cases should remain in a separate group for life. Reports of consecutive examinations provide information on the dynamics of IMI in the individual cow and identify cows with persistent or chronic IMI.

#### Application in herds during a *Streptococcus agalactiae* mastitis outbreak

During an *S. agalactiae* IMI outbreak in a dairy herd, composite milk samples are taken from all lactating cows and quarter secretion samples are obtained from the dry cows not currently under antibiotic treatment. When the heifers presently in late gestation had been reared on fresh milk as calves, they are also sampled because of a risk of them having *S. agalactiae* IMI (Petzer & Karzis 2012).

The initial list of cows that tested positive for *S. agalactiae* is emailed to the producer within 24 hours after receiving the samples at the laboratory. Managers should then immediately separate cows that tested positive for *S. agalactiae*, treat all quarters with of those cows with intra-mammary antimicrobials, and milk these cows last under strict hygiene conditions. Udders of *S. agalactiae* cows should be palpated to identify gross pathology, in which case the cure rate may be low. Any dry cow or heifers positive for *S. agalactiae* IMI are also treated.

After 10–14 days after the last intra-mammary treatment, the *S. agalactiae–*positive cow group is resampled (quarter milk samples) for micro-cytological analysis and composite samples are collected from the rest of the lactating herd. The percentages of *S. agalactiae* cases that are cured, those that persist and the number of new IMI are analysed, and a management plan is formulated based on this information. Cows with *S. agalactiae* IMI are only regarded as being cured when no *S. agalactiae* can be isolated from any of their quarters, when the SCC in all quarters is below 200 000 cells/mL and when there is only a small variation in the SCC between quarters. The IDF-recommended SCC threshold of 200 000 cells/mL is used in this case to be extra cautious not to introduce a cow with persistent *S. agalactiae* IMI again into the negative herd. Depending on parlour hygiene and management, it is possible for one *S. agalactiae*–positive cow to initiate a new outbreak as was experienced in South African herds.

Monitoring of the herd should be continued on a regular basis, and intervals may be monthly or longer until all lactating cows have tested negative for *S. agalactiae* at least two or more consecutive tests. This may take 3–6 months depending on the initial *S. agalactiae* prevalence as well as the motivation and dedication of the producer and milkers (Figure 1).

For most major udder pathogens, different protocols are required. In the case of a *S. uberis* IMI, different strains were identified (Zadoks 2007). Some strains were found to be more likely to cause chronic IMI while others cured spontaneously after a period of only days. Strain typing of *S. uberis* from individual cow samples on a herd basis has not been shown to be cost effective. When *S. uberis* is isolated repeatedly from the same cows, the herd manager is advised to increase the duration of intra-mammary treatment only in the event of clinical mastitis in those cows and to use intra-mammary dry cow therapy at drying-off in those specific cows.

#### Stage of lactation and parity

The immune system of the lactating cow is known to be weakened during the peripartum period because of hormonal changes and many stressors. These can include calving and onset of lactation stress, social stress and stress because of the adaption to a new diet and the onset of a negative energy balance in the cow that can last up to day 100 of lactation (Collard *et al*. 2000). Because of vulnerability of the cow during this period, this stage of the production cycle is critical for monitoring udder health.

#### Udder health up to 30 days post-partum (multiparous and primiparous cows)

The number of new IMI and cases with elevated SCC that occur from 5 to 30 days post-partum provides insight into the udder health during the dry period and calving hygiene. It is also an indication of bacterial cure rate during the dry period. However, there is also a risk of new IMI post-partum when milking commences. Knowledge about the prominent bacterial species causing IMI in a herd enables advisors and managers to identify and deal with the sources and causes of infection timeously and effectively. The report that summarises the species-specific IMI information including total, cured, persistent and new IMI for cows between 5 and 30 days can be used to evaluate udder health during the dry period. The current species-specific IMI and SCC results of individual multiparous cows should be compared to their udder health in their previous late lactation. Results for primiparous cows are indicated in a separate report as total numbers (and percentages) of species-specific IMI that have been isolated. Managers should aim to have less than 10% of cows with IMI and SCC in excess of 200 000 cells/mL during this period and an incidence of less than 5% of clinical mastitis (Bradley 2007; Green & Bradley 2012). An SCC threshold of 200 000 cells/mL is used to be more strict in management decisions of the post-partum group for they may be more susceptible to new IMI because of calving stress, the onset of lactation, social stress and the possibility of developing a negative energy balance (Barkema *et al*. 1999; Fenwick *et al*. 2008).

Heifers are more prone than cows to develop severe udder oedema prior to calving and recently calved primiparous cows are said to have a greater prevalence of mastitis than older cows, despite having less mastitis later in lactation (Barkema *et al*. 1998). Heifers that are close to calving and primiparous cows should receive a diet with an adequate energy balance and without excessive sodium and potassium (Nestor, Hemken & Harmon 1988). Stress around calving should be limited by stimulating heifers to exercise and by avoiding overcrowding in order to reduce negative social interactions (Hutjens & Aalseth 2005). The IMI profile of first lactation cows shortly post-partum is an indication of udder health challenges that have occurred mostly during late pregnancy (environmental bacteria) but can in some cases be traced back to the way they were reared as calves when fed infected milk and kept in groups (Petzer & Karzis 2012).

When bacteria isolated from milk samples shortly post-partum are predominantly contagious, this may indicate treatment failure, the presence of chronic udder damage or new infections contracted during early lactation. When few cases are cured, treatment protocol needs to be revisited to indicate whether the correct antimicrobial product was used for the correct duration of time.

Udders of treated cows should be palpated to determine if there is chronic udder parenchyma damage (fibrosis, nodules or atrophy). A high rate of new IMI caused by contagious bacteria could be an indication of poor milking hygiene of newly calved cows. Trends of total, cured, new and persistent IMI in a herd can be followed over time to improve management decisions.

In the event where most IMI are caused by environmental bacteria shortly post-partum, causes can be chronic infections or treatment failure but new environmental IMI are more likely to have occurred during the dry period or at calving. Most new IMI caused by environmental bacteria are known to occur just after drying-off and shortly before and after calving (Oliver & Sordillo 1988). In these situations, daily pre-calving teat dipping during the high-risk periods in the dry period should be added to the management routine. No teat seal is currently registered on the South African market to assist in the prevention of new IMI during the dry period of cows.

#### Udder Health in early, mid and late lactation (90 days, 180 days and later in lactation)

Bacteriological and SCC results of milk samples during early, mid and late lactation are compared to evaluate progress or failure of udder health management during lactation (Table 4). Important events occur during the first 90 days of lactation, which includes peak milk production and re-breeding.

**TABLE 4:** Herd Udder Health status correlated with different stages of lactation using quarter milk samples.

Diagnosis	Bacteria isolated: Portion of herd	% In early lactation (5–90 days): 34.78	% In mid-lactation (91–180 days): 27.53	% In late lactation (180+ days): 37.67
**Normal**	**None**	**66.68**	**78.97**	**70.22**
**Aseptic mastitis (NSD)**	**None**	**14.58**	**6.57**	**12.50**
Mastitis	*Streptococcus dysgalactiae*	1.04	1.31	0.00
Mastitis	CNS	7.30	2.62	2.87
Mastitis	*Streptococcus uberis*	3.13	1.31	0.96
Mastitis	*Staphylococcus aureus*	2.07	0.00	0.96
**Total % mastitis (M)**	**-**	**13.54**	**5.23**	**4.78**
TCI	CNS	4.17	9.23	12.50
TCI	*Streptococcus uberis*	1.04	0.00	0.00
**Total % (TCI)**	**-**	**5.20**	**9.23**	**12.50**
**Total % with IMIs (M + TCI)**	**-**	**18.75**	**14.46**	**17.28**
**Total % with high SCC (M + NSD)**	**-**	**28.12**	**11.81**	**17.28**

Mastitis criteria: SCC ≥ 300 000 cells/mL and culture positive; NSD criteria: SCC ≥ 300 000 cells/mL and culture negative; TCI criteria: SCC < 300 000 cells/mL and culture positive; Normal criteria: SCC < 300 000 cells/mL and culture negative.

NSD, non-specific disturbance; CNS, coagulase-negative staphylococci, TCI, teat canal infection; IMI, intra-mammary infection; SCC, somatic cell count.

Early lactation is also a high-risk period for metabolic diseases and multifactorial stress together with other diseases such as metritis and mastitis (Bell 1995; Pulfer 1991). The status of IMI in cows at calving will determine udder health in that whole lactation. If recently calved cows have a high incidence of IMI, little progress will be made in lowering the BMSCC, as each animal cured during lactation would be replaced by another infected cow that has recently calved. The management aim should be to have less than 15% cows with SCC in excess of 200 000 cells/mL up to 90 days in lactation (Green & Bradley 2012).

Mid-lactation is generally a lower risk period and a less eventful time for dairy cows than early lactation. During late lactation, the optimum body condition for cows should to be achieved, foetus growth accelerates and cows are prepared for drying-off. The timing of drying-off usually depends on the expected calving date and level of milk yield to allow for a sufficient dry period. Concentrates may need to be reduced when milk yields are still high close to the date of drying-off to prevent excess udder oedema. While there may be a small rise in a cow’s SCC in late lactation, sharp increases that are seen at this stage may be as a result of udder infection or irritation.

#### General udder health of the herd

In the herd used as an example (Table 4), 71.38% of quarters were diagnosed as being normal; 11.6% with NSD, 7.95% with mastitis and 9.06% with TCI. Although the percentage of normal quarters is satisfactory, the 19.55% of quarter with an SCC above 300 000 cells/mL milk is unacceptably high and so are the 17.01% cases of IMI. The quarters with high SCC in the herd originated from 7.95% mastitis and 11.6% NSD cases (Table 4). The main reason for increased SCC is IMI. When no IMI are found in many milk samples with high SCC, the milking machine and stress factors (high THI, overcrowding, mud, nutritional shortcomings and inadequate management) may be indicated as causes (Burvenich *et al*. 2000; Du Preez *et al*. 1990; Sandrucci *et al*. 2014).

The bacteria responsible for IMI in this herd are CNS (13.04%), *S. uberis* (2.14%), *S. aureus* (1.08%) and *Streptococcus dysgalactiae* (0.72%). It was noted that 72.24% of IMIs in early lactation were because of mastitic quarters, while only 27.65% of IMI identified in quarters in late lactation were mastitic (Table 4).

#### Lactation stages: Intra-mammary infections

The portion of cows in early (34.78%), mid (27.53%) and late lactation (37.67%) differ in this herd. The percentages of IMI and high SCC quarters were calculated per lactation period (Table 4). There were IMI in 18.75% of quarters taken from cows in early, 14.46% in mid and 17.28% in late lactation (Table 4). The percentage of IMI detected in early lactation cows was high. This would warrant further investigation into the dry period and calving management, persistence of chronic cases, stress during early lactation and suppressed immunity of cows early in lactation. When the IMI increases with days in milk, a distinction should be made between failure to cure of existing IMI (that increased persistent cases) and an increase of new IMI. Persistent cases may be because of udder parenchyma damage, treatment failure or virulent or resistant pathogen strains and a suppressed host immune system. Depending on the bacterial species involved, new infections may originate from the environment (bedding, pastures, water contamination and inadequate milking machine hygiene), the parlour hygiene during the milking routine or when biosecurity is lacking.

#### Lactation stages: Somatic cell counts

In the example in Table 4 cows in early lactation had the highest percentage (28.10%) quarters with increased SCC compared to mid (11.80%) and late lactation (17.28%) cows, although cows less than 5 days in milk (colostrum) are excluded. Quarters identified with mastitis (13.54%) and NSD (14.58%) contributed almost equally to the high percentage of SCC found in early lactation (Table 4). Therefore, in this herd, suspected causes for both IMI and NSD in early lactating cows needed to be investigated.

### Economic report

Estimations are made on the whole herd and do not take into account variations between cows. The MSD programme is used to estimate milk production losses based on quarter milk sample with elevated SCCs (Giesecke *et al*. 1994; Hortet & Seegers 1998; Sharma *et al*. 2011). The herd used as an example in Table 5 had a daily milk production of 2650 litres. The daily milk loss in this herd because of elevated SCC was estimated to be 126.55 litres. This loss represents an estimated loss of 3796 litres per month and 46 190 litres annually. This represented an estimated loss of 4.77% in potential milk production for this herd.

**TABLE 5:** Estimated milk production losses in a herd associated with elevated quarter milk somatic cell counts of all lactating cows in the herd.

SCC levels × 1000 cells/mL	% Losses based on the SCC level of individual quarters	Number of quarters per SCC level	Loss in litres	Lost revenue
< 125	0.0	183	0.00	R 0.00
125–350	3.7	33	12.26	R 54.54
351–500	11.3	14	15.88	R 70.67
501–750	16.3	8	13.09	R 58.25
> 750	25.0	34	85.32	R 379.68
Inactive quarters	100.0	0	0.00	R 0.00

Estimated daily milk loss: 126.55 litres values at ZAR 563.14; Estimated monthly milk loss: 3796.43 litres values at ZAR 16894.10; Estimated annual milk loss: 46 190 litres values at ZAR 205544.84.

Producer code: 840; Producer: Farmer A; Dairy: Dairy A; Daily milk production: 2650 litres; Milk price per litre: ZAR 4.45; Examination date: 10 September 2015; Selection criteria: All samples tested (*n* = 66 cows).

SCC, somatic cell count.

When making a management decision regarding a *S. agalactiae* IMI in a herd, it is helpful to have an indication of the current production loss because of *S. agalactiae*. In Table 6, the daily milk production loss in the *S. agalactiae*–infected quarters was estimated to be 37.64 litres amounting to an estimated 1.4% production loss. Although this case may not warrant blitz therapy to eradicate *S. agalactiae,* the focus should be on improving the milking routine. This calculation is only based on prevalence at the time of sampling and does not incorporate risks of new infection, shedding, cure rate and the number of persistent cases.

**TABLE 6:** Estimated milk production losses in a herd associated with quarters infected with *Streptococcus agalactiae.*

SCC levels × 1000 cells/mL.	% Losses based on the SCC level of individual quarters	Number of quarters per SCC level	Loss in litres	Lost revenue
< 125	0.0	1	0.00	R 0.00
125–350	3.7	6	2.23	R 10.12
351–500	11.3	4	4.54	R 20.60
501–750	16.3	2	3.27	R 14.86
> 750	25.0	11	27.60	R 125.32
Inactive quarters	100.0	0	0.00	R 0.00

Estimated daily milk loss: 37.64 litres values at ZAR 170.90; Estimated monthly milk loss: 1129 litres values at ZAR 5127.00; Estimated annual milk loss: 13 739 litres values at ZAR 62378.50.

Producer code: 840; Producer: Farmer A; Dairy: Dairy A; Daily milk production: 2650 litres; Milk price per litre: ZAR 4.54; Examination date: 18 April 2015; Selection criteria: Quarters positive for *Streptococcus agalactiae.*

SCC, somatic cell count.

## Conclusion

There are many advantages of having species-specific IMI information about udder health in the current MSD system. It allows early detection of IMI, rapid follow-up on information from tests; there is a short turnaround time after the receipting of milk samples and prompt communication of results to herd managers and owners. The programme firstly allows evaluation of the herd udder health situation enabling the consultant to identify the main causes of udder health problems in detail. This will assist in identifying and eliminating the sources of the problems timeously. At the same time, it provides information on each cow on parity, lactation stage, pregnancy status, production level and mastitis and SCC history to enable informed decisions for individual cows and even individual udder quarters. Management decisions can be based on sound information and cows that are cured or have persistent IMI and new IMI can be identified, based on actual bacterial identification. This improves the accuracy of the decisions made. This approach has proved to be practical and to build the confidence of dairy farm managers (personal experience).
